# Prospective Investigation of ^18^FDG-PET/MRI with Intravoxel Incoherent Motion Diffusion-Weighted Imaging to Assess Survival in Patients with Oropharyngeal or Hypopharyngeal Carcinoma

**DOI:** 10.3390/cancers14246104

**Published:** 2022-12-12

**Authors:** Sheng-Chieh Chan, Chih-Hua Yeh, Shu-Hang Ng, Chien-Yu Lin, Jen-Hung Wang, Joseph Tung-Chieh Chang, Nai-Ming Cheng, Kai-Ping Chang, Jason Chia-Hsun Hsieh

**Affiliations:** 1 Department of Nuclear Medicine, Hualien Tzu Chi Hospital, Buddhist Tzu Chi Medical Foundation, Hualien 970423, Taiwan; 2 School of Medicine, Tzu Chi University, Hualien 970423, Taiwan; 3 Department of Diagnostic Radiology, Linkou Chang Gung Memorial Hospital and Chang Gung University, Taoyuan 333423, Taiwan; 4 Department of Radiation Oncology, Linkou Chang Gung Memorial Hospital and Chang Gung University, Taoyuan 333423, Taiwan; 5 Department of Medical Research, Hualien Tzu Chi Hospital, Buddhist Tzu Chi Medical Foundation, Hualien 970423, Taiwan; 6 Department of Nuclear Medicine, Chang Gung Memorial Hospital, Keelung 204201, Taiwan; 7 Department of Otorhinolaryngology, Linkou Chang Gung Memorial Hospital and Chang Gung University, Taoyuan 333423, Taiwan; 8 Division of Hematology/Oncology, Department of Internal Medicine, Linkou Chang Gung Memorial Hospital and Chang Gung University, Taoyuan 333423, Taiwan

**Keywords:** head and neck neoplasms, intravoxel incoherent motion, magnetic resonance imaging, PET/MRI, positron emission tomography, prognosis

## Abstract

**Simple Summary:**

Intravoxel incoherent motion (IVIM) diffusion-weighted imaging (DWI) is an advanced MRI technique from which both diffusion and perfusion parameters of tumor tissues can be extracted. The question as to whether ^18^F-FDG PET/MRI using the IVIM-DWI technique may provide prognostic information in patients with head-and-neck cancers remains unanswered. This prospective study was therefore designed to evaluate the prognostic significance of IVIM, dynamic contrast-enhanced MRI (DCE-MRI), and PET parameters derived from integrated PET/MRI in patients with oropharyngeal or hypopharyngeal squamous cell carcinomas (OHSCC). Our results indicated that metabolic tumor volume on ^18^F-FDG PET and D* derived from IVIM-DWI were independent risk factors for overall survival, whereas *K^trans^* and iAUC derived from DCE-MRI were significant predictors of recurrence-free survival. Combining PET/MRI biomarkers with clinical risk factors aided in formulating prognostic models, which demonstrated higher accuracy in predicting overall or recurrence-free survival over the traditional TNM staging system.

**Abstract:**

To prospectively investigate the prognostic value of ^18^F-FDG PET/MRI in patients with oropharyngeal or hypopharyngeal squamous cell carcinomas (OHSCC) treated by chemoradiotherapy. The study cohort consisted of patients with OHSCC who had undergone integrated PET/MRI prior to chemoradiotherapy or radiotherapy. Imaging parameters derived from intravoxel incoherent motion (IVIM), dynamic contrast-enhanced MRI (DCE-MRI), and ^18^F-FDG PET were analyzed in relation to overall survival (OS) and recurrence-free survival (RFS). In multivariable analysis, T classification (*p* < 0.001), metabolic tumor volume (*p* = 0.013), and pseudo-diffusion coefficient (*p* = 0.008) were identified as independent risk factors for OS. The volume transfer rate constant (*p* = 0.015), initial area under the curve (*p* = 0.043), T classification (*p* = 0.018), and N classification (*p* = 0.018) were significant predictors for RFS. The Harrell’s c-indices of OS and RFS obtained from prognostic models incorporating clinical and PET/MRI predictors were significantly higher than those derived from the traditional TNM staging system (*p* = 0.001). The combination of clinical risk factors with functional parameters derived from IVIM and DCE-MRI plus metabolic PET parameters derived from ^18^F-FDG PET in integrated PET/MRI outperformed the information provided by traditional TNM staging in predicting the survival of patients with OHSCC.

## 1. Introduction

Oropharyngeal and hypopharyngeal squamous cell carcinomas (OHSCCs) are common head-and-neck cancer (HNC) arising from adjacent anatomic sites. They share both similar lymphatic drainage and treatment approaches. Most patients with OHSCC have aggressive locoregional diseases at presentation [1]. Organ-sparing therapy is generally based on chemoradiotherapy; unfortunately, the outcomes have remained suboptimal, especially for patients with advanced disease, and approximately 65% of patients ultimately develop recurrent or metastatic disease [2]. Since the progression-free survival and overall survival (OS) rates at 3 years are as low as 54% and 63%, respectively [3], the identification of novel clinical-grade prognostic biomarkers is crucial for appropriate treatment decision-making and follow-up strategy.

Traditionally, the two major imaging modalities used for the assessment of patients with HNC are magnetic resonance imaging (MRI) and ^18^F-fluorodeoxyglucose positron emission tomography (^18^F-FDG PET)/computed tomography (CT). However, the combination of MRI with ^18^F-FDG PET (hybrid ^18^F-FDG PET/MRI) has recently gathered remarkable momentum [4,5,6]. In this regard, hybrid PET/MR scanners enable the simultaneous acquisition of excellent soft-tissue contrast (MRI component) and functional metabolic information (PET component). Additionally, the MRI component of PET/MR allows applying functional techniques, namely dynamic contrast-enhanced MRI (DCE-MRI) and diffusion-weighted imaging (DWI), which would improve the prognostic stratification of patients with HNC [3,7,8,9,10,11,12,13].

DCE-MRI provides information regarding blood flow and vascular permeability in the tissues. DWI quantifies the diffusion motion of water molecules by the apparent diffusion coefficient (ADC), which is calculated by a monoexponential model, reflecting cellularity. Notably, the ADC value is influenced not only by the incoherent motion of water due to thermal energy but also by the incoherent motion of water due to microvasculature perfusion. As an extended method based on DWI, intravoxel incoherent motion (IVIM) DWI is an advanced MRI technique that uses multiple b values and bi-exponential fitting equations [14]. This approach does not require the administration of a contrast medium and allows distinguishing true water molecular diffusion from pseudo-diffusion of microcirculation. Specifically, IVIM parameters measure the movements of water molecules—including slow diffusion motion (which is related to cellularity) and fast diffusion movement (which reflects microvascular perfusion). The growing interest in IVIM can largely be attributed to recent advances in hardware and software, and IVIM may have the potential to replace DWI for improved tissue characterization and treatment monitoring [15]. Indeed, IVIM has shown promise in predicting treatment response or prognosis of HNC alone [16,17,18], in combination with DCE-MRI [15,19], and in combination with both DCE-MRI and ^18^F-FDG PET/CT [5,20]. However, there are some inconsistencies in the published literature due to differences in patient characteristics, imaging protocols, treatment regimens, duration of follow-up, and outcome measures.

In contrast to MRI functional parameters, the biomarkers derived from ^18^F-FDG PET can reflect glucose metabolism. Several PET-biomarkers—including standardized uptake value (SUV) or metabolic tumor volume (MTV)—have shown diagnostic and prognostic implications in patients with HNC [3,21,22,23].

Integrated PET/MRI scanners can perform both MRI and PET scanning simultaneously and can, thus, offer various functional and metabolic imaging biomarkers for tumor characteristics at the same time. However, only three previous retrospective studies examined the prognostic utility of multiparametric PET/MRI biomarkers in HNC [5,24,25]. Of the two investigated ADC and PET variables [24,25], the remaining one examined ADC, DCE-MRI, and PET parameters [5]. To our knowledge, there has been no previous attempt to evaluate the prognostic significance of IVIM, DEC-MRI, and PET parameters derived from PET/MRI in patients with OHSCC. This prospective study was undertaken to address this issue.

## 2. Materials and Methods

### 2.1. Patients

Candidates were eligible if they had a biopsy-confirmed diagnosis of OHSCC and no contraindications to MRI scanning. Patients with a history of previous HNC, distant metastases, synchronous second primary malignancies, and renal failure were excluded, as were those unable to provide legally effective consent. The investigation was approved by the Institutional Review Board of the Chang Gung Memorial Hospital (IRB no. 103-7314A3) and conformed with the principles outlined in the Declaration of Helsinki. All participants read and signed an informed consent document prior to beginning the study.

### 2.2. ^18^F-FDG PET/MRI

Patients underwent PET/MRI on an integrated PET/MRI scanner (Biograph mMR, Siemens Healthcare, Erlangen, Germany). After fasting for at least 6 h, patients were injected intravenously with ^18^F-FDG (370 MBq). The examination protocol consisted of whole-body and regional head-and-neck scanning (Table 1). A fast-view T1-weighted MRI localizer sequence was acquired for scout imaging, followed by a Dixon volumetric interpolated breath-hold examination (VIBE) sequence for attenuation correction. Patients then underwent a whole-body PET scanning in 4-bed positions to cover from the head to the proximal thigh. Meanwhile, whole-body MRI acquisition was performed for the corresponding 4-bed positions. Afterward, regional head-and-neck PET was performed with an acquisition time of 10 min, while a dedicated MRI was simultaneously acquired with a T1-weighted Turbo spin echo (TSE) sequence plus a fat-suppressed T2-weighted TSE sequence.

Axial IVIM was then performed using a single-shot spin-echo echo-planar technique with a modified Stejskal-Tanner diffusion gradient pulsing scheme. A total of 10 b-values (0, 20, 40, 80, 100, 200, 400, 800, 1200, and 1500 s/mm^2^) were applied. Thereafter, DCE-MRI was performed using a 3D T1-weighted spoiled gradient-echo sequence. Baseline longitudinal relaxation time (T1_0_) values were calculated from images acquired with four different flip angles (4°, 8°, 15°, and 25°). After intravenous injection of the paramagnetic contrast agent (infusion rate: 3 mL/s), we applied the same pulse sequence (flip angle: 15°) to acquire the dynamic series. Finally, contrast-enhanced regional head-and-neck and whole-body MRI was performed subsequently. The total acquisition time was approximately 42 min, whereas the average in-room time for PET/MR was ~56 min. PET data were reconstructed using an ordinary Poisson ordered-subset expectation maximization with three iterations, 21 subsets, and a 4-mm Gaussian post-processing filter into 344 × 344 matrices.

### 2.3. Analysis of Image

We utilized the PMOD 4.2 software (PMOD Technologies Ltd., Zurich, Switzerland) to conduct tumor segmentation in PET images. Using a threshold SUV of 2.5 [26,27], tumor boundaries within the regions of interest (ROIs) were drawn in the axial, coronal, and sagittal PET planes after the exclusion of areas characterized by physiological FDG uptake. Primary tumor SUV, MTV, and total lesion glycolysis (TLG) were determined automatically.

DCE-MRI images were analyzed with dedicated software (Tissue 4D, Siemens Medical Systems, Erlangen, Germany). T1 maps were automatically obtained following registration of pre- and post-contrast images and motion correction. A population-based approach was applied to determine the arterial input function after scaling according to the gadolinium dose [28]. Pharmacokinetic parameters—including the volume transfer constant (*K^trans^*), rate constant (*K_ep_*), and extracellular space leakage (*V_e_*)—were determined from each ROI within the primary tumor area, and the initial area under the enhancement curve (iAUC) was calculated. *K^trans^* was defined as the transfer constant between blood plasma and extravascular extracellular blood space (EES), whereas *K_ep_* expresses the rate constant between EES and blood plasma. *V_e_* is an expression of fractional EES volume. In this study, we defined the iAUC as the initial area under the time-concentration curve calculated on post-enhancement images within the first 60 s. Freehand ROIs were manually drawn in the primary tumor area by an experienced head and neck radiologist; necrotic and hemorrhagic foci were carefully excluded.

IVIM maps were generated by fitting the IVIM data obtained with 10 b-values (0, 20, 40, 80, 100, 200, 400, 800, 1200, and 1500 s/mm^2^) to the bi-exponential model expressed by the equation *S_b_/S*_0_ = (1 − *f*)*e*^−*bD*^ + *fe^−b(D^* ^+ D*)^, where S_b_ and S_0_ are the signal intensities in the diffusion gradient factors of b and 0, respectively, *f* is the perfusion fraction, *D*, the slow diffusion coefficient, and D*, the pseudo-diffusion coefficient. Additionally, ADC maps were generated by fitting the IVIM data obtained with 2 b-values (0, 800 s/mm^2^) to the conventional mono-exponential model expressed by the equation *S_b_ = S*_0_ *× e*^−*b*ADC^. ROIs were also manually drawn by an experienced head and neck radiologist in the primary tumor area on IVIM maps and ADC maps, respectively, in a manner similar to that described for the DCE-MRI analysis. Thereafter, the mean *D*, D*, *f*, and ADC values of the primary tumor area were subsequently extracted for further analysis.

### 2.4. Treatment and Follow-Up

Intensity-modulated radiotherapy or volumetric-modulated radiotherapy was delivered using 6 mega-voltage photon beams. We used the simultaneously-integrated boost or multi-scale shrinkage-field techniques for a total of 33–36 treatment fractions. The prophylactic dose was 46–50 Gy to the gross tumor area and the entire neck, followed by a cone-down boost at 70–72 Gy to the gross tumor area and grossly involved lymph nodes. While patients with stage 1 or 2 diseases received definitive radiotherapy, those with a tumor stage of 3 or higher were treated with concurrent chemoradiotherapy. Concurrent chemoradiotherapy consisted of intravenous cisplatin 50 mg/m^2^ on day 1 and oral tegafur 800 mg/day plus oral leucovorin 60 mg/day from day 1 to day 14. The scheme was repeated biweekly throughout the entire radiotherapy course [29]. The follow-up schedule comprised clinical examinations every 1–3 months. Patients underwent MRI three months after the completion of chemoradiotherapy. MRI or CT was then performed every 6 months or as needed in the presence of worsening clinical conditions.

### 2.5. Statistical Analysis

Sample size estimation was calculated with the following formula: N = 10 × k/p, where p is the smallest of the proportions of positive cases in the population and k is the number of covariates (i.e., the number of independent variables). In our study, the proportion for overall survival (OS) was approximately 0.47 (i.e., *p* = 67/144 = 0.47), and the number of independent variables in the Cox regression model for OS was 6 (i.e., k = 6). The minimum sample size of the population was, therefore, 128 (N = 10 × 6/0.47). Under the hypothesis of a 10% drop-out rate, it would have been necessary to include at least 142 patients. To ensure data reliability, a total of 144 patients were included for analysis. The main study endpoints were OS and recurrence-free survival (RFS). OS was defined as the time elapsed from the date of diagnosis to the date of death, whereas RFS was the time between the end of chemoradiotherapy and the day of tumor recurrence. Censoring was performed on the date of the last follow-up (i.e., administrative censoring). Serial cutoff points were tested for each imaging biomarker, and optimal values were defined as those characterized by the lowest *p*-values. Survival curves were plotted with the Kaplan-Meier method and compared with the log-rank test. We initially used univariate regression analysis to evaluate the association between each risk factor and the outcomes of interest. To explore independent association, we applied multivariable Cox regression models. The associations between the PET/MRI parameters were investigated using Pearson’s correlation coefficient. Analyses were carried out in SPSS (version 21.0, IBM, Armonk, NY, USA), and statistical significance was determined using a 2-tailed *p*-value < 0.05. Subsequently, prognostic models were devised based on clinical risk factors and PET/MRI biologic imaging biomarkers. The prognostic accuracy of each prediction tool was assessed using Harrell’s concordance index (c-index) [30].

The models were internally validated using the bootstrapping method calculated from 1000 bootstrap samples drawn from the original sample with replacement. Bootstrapping was performed using R (version 3.4.2; R Foundation for Statistical Computing, Vienna, Austria). The training cohort consisted of all our patients available for analysis (*n* = 144).

## 3. Results

**Figure 1:**
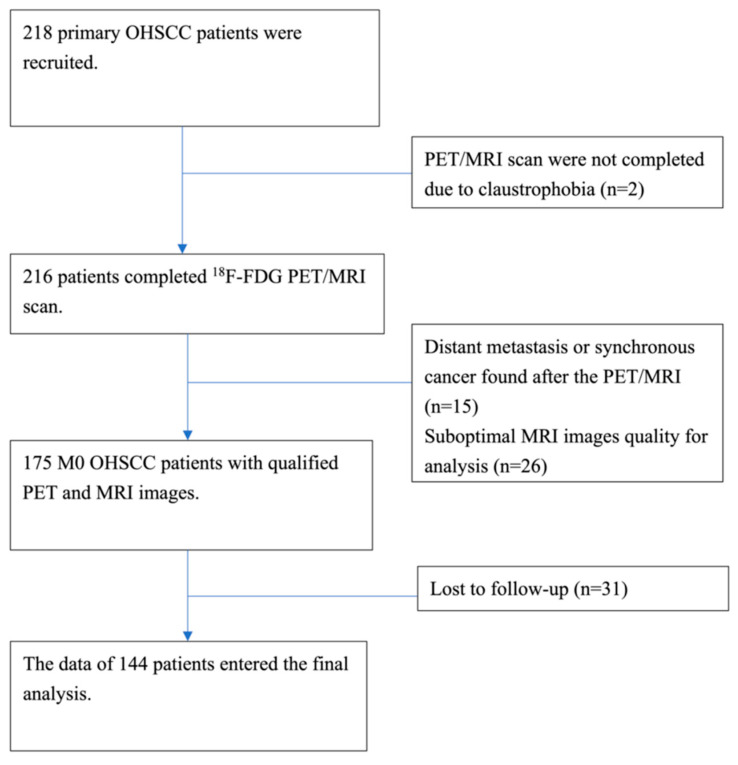
Study flowchart. OHSCC: oropharyngeal and hypopharyngeal squamous cell carcinoma.

Between August 2015 and October 2018, a total of 218 potentially eligible patients were consecutively identified. All were treatment-naïve and had a histological diagnosis of primary OHSCC. After the exclusion of patients with distant metastases or synchronous cancers (*n* = 15), suboptimal image quality due to small tumor size or artifacts (*n* = 26), loss to follow-up (*n* = 31), or inability to complete PET/MRI imaging due to claustrophobia (*n* = 2), the final study cohort consisted of 144 patients (Figure 1). Table 2 summarizes the general characteristics of the study participants.

### 3.1. Univariate and Multivariable Predictors of Survival Outcomes

The median follow-up time for the entire study cohort was 35.8 months (range 2.0–70.3 months), whereas surviving patients were followed up for a median of 47.0 (range 22.3–70.3 months). At the time of analysis, 67 (47%) patients were dead, and 61 (42%) patients had recurrent tumors. The 3-year RFS and OS rates in the study patients were 49.7% and 55.9%, respectively.

**Figure 2:**
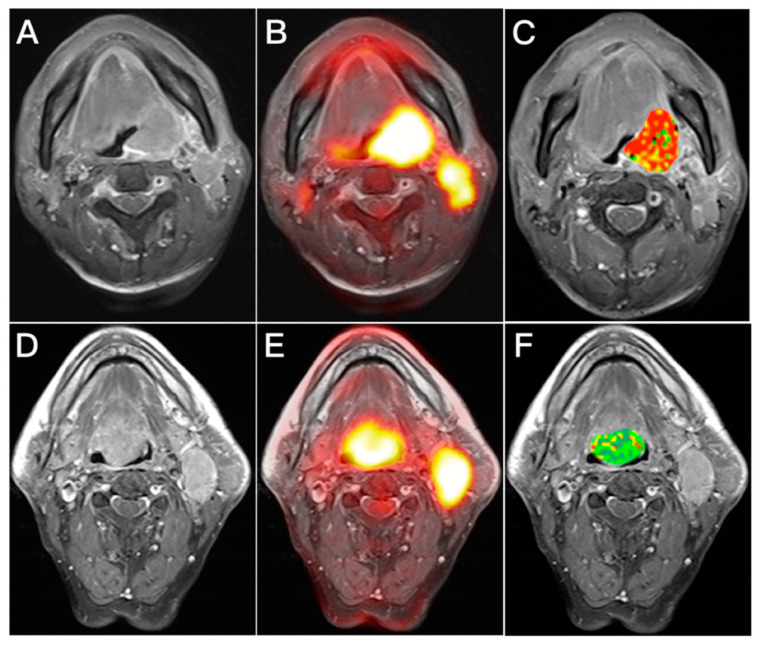
A combination of PET metabolic and IVIM functional biomarkers aided in predicting overall survival. (**A**–**C**) Representative axial images of a primary left tonsillar oropharyngeal cancer patient with T3N2b disease. (**A**) Contrast-enhanced MR image; (**B**) ^18^F-FDG PET/MRI image; and (**C**) Contrast-enhanced MR image with an overlaid D* map of the primary tumor. This patient had a high MTV of 111 mL and a high D* value of 481 × 10^−3^ mm^2^/s. This patient died of tumor recurrence with a short overall survival period of 8 months after chemoradiotherapy. (**D**–**F**) Axial PET and MRI images of a primary tongue base oropharyngeal carcinoma patient with T3N2b disease. (**D**) Contrast-enhanced T1-weighted MR image; (**E**) ^18^F-FDG PET/MRI image; and (**F**) Contrast-enhanced MR image with an overlaid D* map of the primary tumor. He had a low MTV of 58 mL and a low D* value of 255 × 10^−3^ mm^2^/s. He survived without disease recurrence for 4 years after chemoradiotherapy. MTV: metabolic tumor volume.

**Figure 3:**
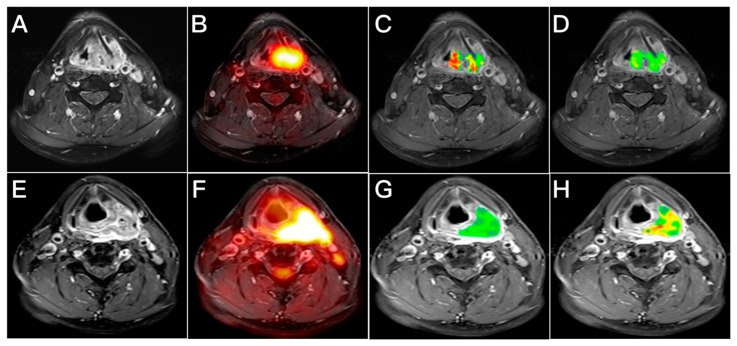
Integrating TN classification with MRI perfusion biomarkers aided in predicting recurrence-free survival. (**A**–**D**) Representative axial images of a primary hypopharyngeal cancer patient with T4aN0 disease. (**A**) Contrast-enhanced MR image; (**B**) ^18^F-FDG PET/MRI image; (**C**) Contrast-enhanced MR image with an overlaid *K^trans^* map of the primary tumor; and (**D**) Contrast-enhanced MR image with an overlaid iAUC map of the primary tumor. This patient had a high *K^trans^* value of 416 × 10^−3^ min^−1^ and a low iAUC value of 424. This patient had local tumor recurrence 12 months after chemoradiotherapy. (**E**–**H**) Representative axial images of a primary hypopharyngeal cancer patient with T4aN2b disease. (**E**) Contrast-enhanced MR image; (**F**) ^18^F-FDG PET/MRI image; (**G**) Contrast-enhanced MR image with an overlaid *K^trans^* map of the primary tumor; and (**H**) Contrast-enhanced MR image with an overlaid iAUC map of the primary tumor. He had a low *K^trans^* value of 117 × 10^−3^ min^−1^ and a high iAUC value of 1044. This patient survived without disease recurrence 5 years after chemoradiotherapy.

In univariate analysis (Table 3), tumor stage, T classification, N classification, maximum SUV, MTV, TLG, and D* were identified as significant risk factors for OS, whereas hemoglobin level, T classification, N classification, tumor stage, MTV, TLG, *K^trans^*, *K_ep_*, *V_e_*, iAUC, and *f* were significantly associated with RFS. After adjustment for potential confounders in multivariable analysis (Table 4), T classification (*p* < 0.001), MTV (*p* = 0.013), and D* (*p* = 0.008) retained their independent prognostic significance for OS (Table 4), whereas advanced T classification (*p* = 0.018), high N classification (*p* = 0.018), *K^trans^* > 0.298 min^−1^ (*p* = 0.015), and iAUC ≤ 1007.2 (*p* = 0.043) were independently associated with RFS. Figure 2 and Figure 3 show illustrative cases characterized by different IVIM and DCE-MRI PET/MRI parameters and present their survival outcomes.

### 3.2. Performance of Multiparametric Prognostic Models Comprising IVIM PET/MRI Biomarkers

We finally devised multiparametric prognostic models that combined PET/MRI biomarkers and clinical variables identified as independent risk factors in multivariable analysis. A covariate was assigned a value of 0 when absent or 1 when present. Harrell’s c-indices of the prognostic models are listed in Table 5. The Harrell’s c-indices of OS and RFS obtained from multiparametric PET/MRI models were significantly higher than those from the traditional tumor staging system (OS: 0.70 vs. 0.60, *p* = 0.001; RFS: 0.68 vs. 0.58, *p* = 0.001, respectively). When the models were internally validated using bootstrapping, the c-indices of the multiparametric PET/MRI prognostic models comprising PET/MRI biomarkers were comparable to those observed in the training cohort (Supplementary Table S1).

### 3.3. Correlation between Imaging Parameters

Correlation analysis of PET and MRI functional parameters were demonstrated in Supplementary Table S2. We found strong positive correlations between ADC and *D* derived from the IVIM image (*r* = 0.942) as well as between *K^trans^* and *K_ep_* from the DCE-MRI (*r* = 0.865). The correlations between MTV and D*, as well as *K^trans^* and iAUC, were weak (*r* = −0.113 and 0.263, respectively).

## 4. Discussion

The results of this prospective study demonstrate the clinical feasibility of a single ^18^F-FDG PET/MRI examination to simultaneously obtain IVIM-, DCE-MRI-, and PET-derived parameters. These imaging biomarkers provide complementary information on the viability and metabolic activity of tumor cells and may therefore improve the prognostic stratification of patients with malignancies [31]. On analyzing a cohort of patients with OHSCC, we identified T classification, MTV, and D* as independent risk factors for OS, whereas *K^trans^*, iAUC, T classification, and N classification were independent predictors for RFS. Intriguingly, the combination of clinical risk factors with functional parameters derived from IVIM and DCE-MRI plus metabolic parameters derived from ^18^F-FDG PET in integrated PET/MRI outperformed the information provided by traditional TNM staging in predicting the survival of patients with OHSCC after chemoradiotherapy. These findings may have clinical implications by identifying patients harboring adverse prognostic factors who are more likely to benefit from aggressive therapeutic approaches. This would ultimately make the treatment of OHSCC more personalized and facilitate early detection of potentially salvageable lesions.

In this study, we found that D*—a perfusion-related IVIM parameter—had a significant predictive value for OS, whereas *K^trans^* and iAUC—two perfusion parameters derived from DCE-MRI—independently predicted RFS. While both DCE-MRI- and IVIM-derived perfusion parameters reflect tumor vascularity, they exhibit different aspects of tumor vessels. Specifically, DCE-MRI-derived perfusion parameters are reflective of microvascular density, while IVIM-derived perfusion parameters primarily quantify microscopic translational motions related to the blood microcirculation within randomly distributed capillaries [32]. As seen in this study, IVIM perfusion parameters could not be replaced with DCE-MRI perfusion parameters. Instead, they appeared to be complementary, and the multiparameter PET/MRI prognostic models combining IVIM, DCE-MRI, and PET biomarkers may comprehensively capture different biological underpinnings of OHSCC, ultimately improving prognostic prediction.

Integrated ^18^F-FDG PET/MRI can provide simultaneous functional and metabolic information at the same bed position that would be more accurate than the data obtained by separate PET/CT, and MRI scans at different time intervals with inconsistent head-and-neck positions. However, only a limited number of previous retrospective studies have examined the predictive value of combined functional and metabolic parameters derived from ^18^F-FDG PET/MRI in patients with HNC [5,24,25]. Kim et al. [25] analyzed PET and DWI parameters in a cohort of 72 patients with HNC. They found that MTV and MTV/ADC were independent prognostic factors for disease-free survival. Kedves et al. [24] examined DWI and PET metabolic parameters in 68 patients with HNC. While ADC did not predict therapeutic response, several PET parameters—including SUV, SUV normalized to lean body mass (SUL), MTV, and TLG—showed significant associations with treatment outcomes. In an analysis of DWI-, DCE-MRI-, and PET-derived parameters in 45 patients with locally advanced OHSCC, Pace et al. [5] found that only SUL was the independent prognostic factor for OS. In this prospective ^18^F-FDG PET/MRI study of 144 patients of OHSCC, we investigated IVIM in addition to DCE-MRI and PET parameters. This is, to our knowledge, the first prospective study to investigate IVIM, DCE-MRI, and PET parameters from simultaneous ^18^F-FDG PET/MRI for the prediction of survival in patients with OHSCC treated with chemoradiotherapy, and we documented that D* (IVIM parameter), *K^trans^* and iAUC (DCE-MRI parameters), and MTV (PET parameter) were significant predictive imaging biomarkers.

Previously published studies in patients with HNC have investigated the prognostic value of IVIM alone [16,17,18], in combination with DCE-MRI [15,19], and in combination with both DCE-MRI and ^18^F-FDG PET/CT [20,33], albeit with discrepant results. Three parameters, including *D*, D*, and *f*, can be derived from the bi-exponential fitting of IVIM data obtained with multiple different b-values. *D* is a pure diffusion coefficient and is inversely correlated with tumor cellularity. Tumors with low cellularity due to necrosis or apoptosis have been reported to be more chemo- and radioresistant [33]. A high pretreatment D value has been shown to have an adverse prognostic significance in HNC [16,19]. However, data have been inconsistent with other studies showing either opposite results [20] or null findings [15,17,18,33]. In the present study, we found that pretreatment *D* and ADC values did not show a significant association with the study endpoints. These results suggest that water diffusivity—regardless of its quantification by the *D* value derived from a bi-exponential IVIM model or by the ADC value derived from a conventional mono-exponential DWI model—might have no prognostic significance in OHSCC.

The D* value is the pseudo-diffusion coefficient representing the perfusion-related incoherent microcirculation of the tumor. The *f* value is the microvascular volume fraction flowing into the capillaries that reflects neovascularity. Favorable outcomes have been previously reported in HNC patients with high D* [15,16] or f values [15,20], which may be due to the fact that increased perfusion improves delivery of chemotherapeutic drugs and increases oxygen tension within the tumor, resulting in good treatment response and local control. Our study supported that high *f*-values were associated with prolonged survival. However, there have been other reports showing poor outcomes in patients with HNC and high *f* [17,18] values. A high micro-vessel volume fraction may be associated with a higher likelihood of nodal and distant metastases, leading to an adverse prognosis for survival in these reports [34,35]. In this cohort of OHSCC patients, *f* was a prognostic factor in univariate analysis for OS but lost its significant prognostic value in the multivariate model. We thought that *f* might be overwhelmed by *K^trans^* (vascular permeability) relating to accelerated imbalanced angiogenesis with resultant high vascular leakage and greater resistance to radiotherapy. This ^18^F-FDG PET/MRI study showed that D* was an independent imaging predictor for OS other than MTV, but there was no significant correlation between them. Therefore, they would likely have a synergic effect in predicting OS; indeed, the multiparametric PET/MRI model with a combination of D* and MTV did show high prognostic value. However, further prospective studies focusing on the use of IVIM combined with PET on specific tumor sites are needed to allow more definite conclusions, particularly by using PET/MRI that would avoid the confounding factor of different times of data acquisition and different head positions attained separately from individual MRI and PET/CT scanners.

There are limitations to this study. First, the human papillomavirus (HPV) infection status was not determined in all patients. While this study commenced in 2015, we did not routinely investigate the presence of HPV infections until the release of the eighth edition of the American Joint Committee on Cancer staging system (2017). The expression of p16 was not entered into univariate or multivariate analyses to avoid selection bias. Larger prospective cohort studies are required to separately investigate the prognostic impact of PET/MRI parameters in patients with oropharyngeal versus hypopharyngeal cancer. Second, this is a single-center study, and our results require independent validation in larger multi-center investigations. Third, an SUV of 2.5 was used for tumor contouring. There is still no consensus regarding the optimal methodology for PET image segmentation in HNC. However, our method is in line with several previous studies in the field [26,27,36,37], allowing direct comparisons with the published literature. Fourth, our model might not be applicable to all patients with OHSCC, particularly in the presence of small-sized/cystic tumors or susceptibility artifacts. Further refinements of functional imaging technology are necessary before routine clinical application.

## 5. Conclusions

This prospective study demonstrates that obtaining IVIM, DCE-MRI, and PET imaging biomarkers from ^18^F-FDG PET/MRI in patients with primary OHSCC is feasible. MTV derived from ^18^F-FDG PET and D* derived from IVIM were independent risk factors of OS, while *K^trans^* and iAUC—both derived from DCE-MRI—were identified as significant predictors of RFS. The combination of clinical risk factors with functional parameters derived from IVIM and DCE-MRI plus metabolic parameters derived from ^18^F-FDG PET in integrated PET/MRI outperformed the information provided by traditional TNM staging in predicting the survival of OHSCC patients treated with chemoradiotherapy (c-index for OS = 0.70 vs. 0.60, respectively, *p* = 0.001). Our results have highlighted a novel application of PET/MR-derived biomarkers in the field of oncology and may pave the way to future longitudinal studies aimed at determining their predictive values in other malignancies of the head and neck. Further research should also focus on the combined use of IVIM and PET/MRI to confirm and expand our current data.

## Figures and Tables

**Table 1:** MRI sequence parameters used for integrated PET/MRI.

Contrast	Region	Sequence	TR	TE	ST	FOV	VS	PAT	T
Pre-contrast	Whole body	Dixon VIBE AC	3.6	1.23		500	4.1 × 2.6 × 3.1	2	01:35
	Whole body	Ax T2 HASTE	1000	84	6	380	1.5 × 1.2 × 6.0	2	03:00
	Whole body	Cor STIR	1000	51	6	450	2.5 × 1.8 × 6.0	3	03:55
	Whole body	Sag STIR	3400	57	4	260	1.4 × 1.0 × 4.0	2	04:25
	Whole body	Sag T1	450	9.8	4	280	1.5 × 1.1 × 4.0	2	04:06
	Head Neck	Dixon VIBE AC	3.6	1.23		500	4.1 × 2.6 × 3.1	2	00:19
	Head Neck	Cor T1 TSE	528	12	4	300	1.2 × 0.9 × 4.0	2	01:28
	Head Neck	Cor T2 TSE FS	4300	83	4	300	1.1 × 0.9 × 4.0	2	02:45
	Head Neck	Ax T1 TSE	580	11	4	200	0.9 × 0.8 × 4.0	2	01:32
	Head Neck	Ax T2 TSE FS	5730	87	4	200	0.8 × 0.6 × 4.0	2	03:11
	Head Neck	Ax IVIM (10 b-values)	2900	79	4	240	2.0 × 2.0 × 5.0	2	05:34
Post-contrast	Head Neck	Ax DCE MRI	3.73	1.16	5	256	2.0 × 2.0 × 5.0	2	05:04
	Head Neck	Cor T1 TSE FS	679	11	4	300	1.2 × 0.9 × 4.0	2	01:53
	Head Neck	Ax T1 TSE FS	520	9.7	4	200	0.8 × 0.6 × 4.0	2	02:14
	Whole body	Ax T1 VIBE FS	4.56	1.95	3	400	1.9 × 1.4 × 3.0	2	01:12

TR, repetition time in ms; TE, echo time in ms; ST, Slice thickness in mm; FOV, field of view in mm; VS, voxel size in mm; PAT, parallel acquisition technique; T, scanning time in min; VIBE, volumetric interpolated breath-hold examination; AC, attenuation correction; HASTE, half-Fourier single-shot turbo spin-echo; STIR, short tau inversion recovery; TSE, turbo spin-echo; FS, fat saturation; IVIM, intravoxel incoherent motion.

**Table 2:** General characteristics of the study participants (*n* = 144).

Variable	Number of Patients (%)
Age (years), mean ± SD	60 ± 10
Sex	
Male	134 (93)
Female	10 (7)
Tumor site	
Oropharynx	70 (49)
Hypopharynx	74 (51)
Tumor stage	
I	7 (5)
II	21 (15)
III	30 (20)
IVa-b	86 (60)
T classification	
T1	6 (4)
T2	37 (26)
T3	18 (12)
T4	83 (58)
N classification	
N0	30 (21)
N1	12 (8)
N2	87 (60)
N3	15 (11)
Hemoglobin (g/dL), mean ± SD	14 ± 1.9
Smoking	
Yes	120 (83)
No	24 (17)
Alcohol drinking	
Yes	120 (83)
No	24 (17)
Expression of p16	
Positive	15
Negative	67
Unavailable	62

Data are expressed as counts and percentages (in parentheses) unless otherwise indicated. SD, standard deviation.

**Table 3:** Univariate analysis of clinical factors and PET/MRI biological imaging markers in relation to overall survival and recurrence-free survival in patients with oropharyngeal or hypopharyngeal squamous cell carcinoma.

Variable	Number of Patients	Overall Survival		Recurrence-Free Survival	
		3-Year OS (Number of Events)	*p*-Value	3-Year RFS (Number of Events)	*p*-Value
Age (years)			0.427		0.108
≤60	76	53.5 (39)		44.8 (40)	
>60	68	58.6 (28)		57.5 (26)	
Sex			0.144		0.481
Male	134	54.0 (65)		60.0 (4)	
Female	10	80.0 (2)		50.0 (62)	
Tumor site			0.756		0.834
Oropharynx	70	56.3 (34)		51.2 (33)	
Hypopharynx	74	55.3 (33)		50.2 (33)	
Tumor stage			<0.001		0.018
I-II	28	89.1 (4)		74.1 (9)	
III-IV	116	47.9 (63)		44.4 (57)	
T classification			<0.001		<0.001
T1-2	43	85.8 (7)		75.9 (12)	
T3-4	101	43.0 (60)		38.5 (54)	
N classification			0.014		0.002
N0-1	42	73.7 (11)		75.1 (10)	
N2-3	102	48.9 (56)		41.3 (56)	
Hemoglobin (g/dL)			0.103		0.019
≤13.9	72	49.4 (38)		41.4 (39)	
>13.9	72	62.4 (29)		59.9 (27)	
Smoking			0.246		0.185
No	24	66.7 (8)		61.6 (8)	
Yes	120	53.8 (59)		48.7 (58)	
Alcohol consumption			0.173		0.928
No	24	66.2 (8)		49.1 (12)	
Yes	120	53.9 (59)		51.0 (54)	
*Imaging Biomarker*					
SUV_max_			0.001		0.130
≤14.2	59	70.9 (17)		57.0 (25)	
>14.2	85	45.5 (50)		46.2 (41)	
MTV (mL)			<0.001		0.001
≤81.6	109	64.7 (41)		57.4 (44)	
>81.6	35	28.6 (26)		29.1 (22)	
TLG (g/mL × mL)			0.001		0.005
≤464.5	109	64.0 (42)		56.4 (45)	
>464.5	35	31.4 (25)		32.1 (21)	
*K^trans^* (10^−3^ min^−1^)			0.350		0.034
≤297.8	117	56.8 (53)		54.2 (51)	
>297.8	27	50.9 (14)		34.2 (15)	
*K_ep_* (10^−3^ min^−1^)			0.096		0.039
≤241.3	110	58.7 (48)		54.7 (48)	
>241.3	34	46.4 (19)		35.9 (18)	
*V_e_* (10^−3^)			0.993		0.007
≤122.3	23	55.8 (10)		28.7 (15)	
>122.3	121	55.8 (57)		54.9 (51)	
iAUC			0.780		0.024
≤1007.2	126	55.1 (59)		47.0 (63)	
>1007.2	18	61.1 (8)		79.3 (3)	
ADC_mean_ (10^−3^ mm^2^/s)			0.300		0.599
≤1389	117	57.0 (53)		49.7 (56)	
>1389	27	51.6 (14)		56.3 (10)	
D* (10^−3^ mm^2^/s)			0.012		0.093
≤403.8	41	70.6 (12)		60.3 (15)	
>403.8	103	49.9 (55)		46.9 (51)	
*D* (10^−3^ mm^2^/s)			0.146		0.947
≤1239.9	122	57.7 (49)		51.2 (52)	
>1239.9	32	49.2 (18)		48.9 (14)	
*f* (%)			0.070		0.022
≤165.1	97	49.0 (51)		43.6 (52)	
>165.1	47	70.1 (16)		66.3 (14)	

OS, overall survival; RFS, recurrence-free survival; TLG, total lesion glycolysis; MTV, metabolic tumor volume; SUV_max_, maximum standardized uptake value; ADC, apparent diffusion coefficient; *K*^trans^, volume transfer constant; *K*_ep_, flux rate constant; *V*_e_, extracellular volume ratio; iAUC, initial area under the curve; *D*, diffusion coefficient; D*, pseudo-diffusion coefficient; *f*, perfusion fraction.

**Table 4:** Multivariable analysis of clinical risk factors and PET/MRI biological imaging markers in patients with oropharyngeal or hypopharyngeal squamous cell carcinoma.

Variable	Multivariate Analysis			
	Overall Survival		Recurrence-Free Survival	
	Hazard Ratio (95% CI)	*p*-Value	Hazard Ratio (95% CI)	*p*-Value
Tumor stage		ns		ns
T classification	4.571 (2.043–10.224)	<0.001	2.187 (1.144–4.184)	0.018
N classification		ns	2.343 (1.158–3.861)	0.018
Hemoglobin	-	-		ns
SUV_max_		ns	-	-
MTV	1.907 (1.149–3.165)	0.013		ns
*K^trans^*	-	-	2.114 (1.158–3.861)	0.015
*K_ep_*	-	-		ns
*V_e_*	-	-		ns
iAUC	-	-	0.297 (0.092–0.962)	0.043
D*	2.331 (1.243–4.368)	0.008	-	-
*f*	-	-		ns

CI, confidence interval; MTV, metabolic tumor volume; SUV_max_, maximum standardized uptake value; ADC_,_ apparent diffusion coefficient; *K*^trans^, volume transfer constant; *K*_ep_, flux rate constant; *V*_e_, extracellular volume ratio; iAUC, initial area under the curve; D*, pseudo-diffusion coefficient; *f*, perfusion fraction; ns, not significant.

**Table 5:** Comparison of Harrell’s concordance indices between tumor stage and prognostic models based on clinical factors and PET/MRI biomarkers.

Variable	Overall Survival		Recurrence-Free Survival	
	Concordance Index	95% CI	Concordance Index	95% CI
Tumor stage	0.60	0.56–0.64	0.58	0.53–0.62
PET/MRI prognostic model for OS	0.70 *	0.64–0.76	-	-
PET/MRI prognostic model for RFS	-	-	0.68 **	0.62–0.74

* *p* = 0.001 in comparison with tumor stage; ** *p* = 0.001 in comparison with tumor stage; CI, confidence interval; OS, overall survival; RFS, recurrence-free survival.

## Data Availability

The data presented in this study are available on request from the corresponding author.

## Supplementary Materials

The following supporting information can be downloaded at: https://www.mdpi.com/article/10.3390/cancers14246104/s1, Table S1: Prognostic performance of the tumor stage and PET/MRI prognostic models in the training and validation cohorts, Table S2: Correlation analysis of the PET/MR parameters.
