# Supplementary material for: Prospective Investigation of 18FDG-PET/MRI with Intravoxel Incoherent Motion Diffusion-Weighted Imaging to Assess Survival in Patients with Oropharyngeal or Hypopharyngeal Carcinoma
Source: Cancers (Basel). 2022 Dec 12;14(24):6104. doi: 10.3390/cancers14246104 (PMC9775681; doi:10.3390/cancers14246104)
Supplement: Supplementary file 1 [file cancers-2017589-supplementary.pdf › cancers-2017589-supplementary.pdf]

Supplementary Table S1. Prognostic performance of the tumor stage and PET/MRI prognostic models in the training and validation cohorts.

	Training Cohort				Validation Cohort			
	Overall Survival		Recurrence-Free Survival		Overall Survival		Recurrence-Free Survival	
	c-index	95% CI	c-index	95% CI	c-index	95% CI	c-index	95% CI
Tumor stage	0.60	0.56–0.64	0.58	0.53–0.62	0.60	0.56–0.64	0.57	0.53–0.62
PET/MRI prognostic model for OS	0.70	0.64–0.76	-	-	0.70	0.64–0.76	-	-
PET/MRI prognostic model for RFS	-	-	0.68	0.62–0.74	-	-	0.69	0.63–0.74

CI, confidence interval; OS, overall survival; RFS, recurrence-free survival.

Supplementary Table S2. Correlation analysis of the PET/MR parameters.

Parameter	SUVmax	MTV	ADC	K^{trans}	K_{ep}	V_e	iAUC	D^*	D	f
SUVmax	1.000									
MTV	0.416 (<0.001)	1.000								
ADC	−0.171 (0.040)	−0.138 (0.099)	1.000							
K^{trans}	−0.034 (0.688)	−0.013 (0.876)	−0.013 (0.876)	1.000						
K_{ep}	−0.007 (0.938)	0.001 (0.986)	−0.118 (0.158)	0.865 (<0.001)	1.000					
V_e	−0.159 (0.057)	−0.079 (0.348)	0.271 (0.001)	0.050 (0.549)	−0.165 (0.048)	1.000				
iAUC	0.035 (0.681)	−0.078 (0.354)	0.189 (0.023)	0.263 (0.001)	−0.041 (0.629)	0.475 (<0.001)	1.000			
D^*	−0.082 (0.328)	−0.113 (0.178)	0.136 (0.105)	−0.006 (0.945)	0.089 (0.288)	0.052 (0.540)	−0.023 (0.782)	1.000		
D	−0.139 (0.097)	−0.098 (0.243)	0.942 (<0.001)	−0.050 (0.550)	−0.138 (0.099)	0.244 (0.003)	0.177 (0.034)	0.147 (0.079)	1.000	
f	−0.128 (0.126)	−0.240 (0.004)	0.441 (<0.001)	0.022 (0.793)	−0.007 (0.934)	0.008 (0.927)	0.049 (0.559)	0.311 (<0.001)	0.227 (0.006)	1.000

Data are expressed as correlation coefficients (Pearson *r*) and *p* values; *r* (*p* value)
